# Transcriptome analysis of dormant tomonts of the marine fish ectoparasitic ciliate *Cryptocaryon irritans* under low temperature

**DOI:** 10.1186/s13071-016-1550-1

**Published:** 2016-05-13

**Authors:** Fei Yin, Peng Sun, Jiteng Wang, Quanxin Gao

**Affiliations:** Key Laboratory of East China Sea and Oceanic Fishery Resources Exploitation, Ministry of Agriculture, East China Sea Fisheries Research Institute, Chinese Academy of Fishery Sciences, Room 316, Building 6, 300 Jungong Road, Shanghai, 200090 PR China; Fisheries College of Zhejiang Ocean University, Zhoushan, Zhejiang 316022 PR China

**Keywords:** *Cryptocaryon irritans*, Transcriptome, Dormant tomont, Cell division, Low temperature

## Abstract

**Background:**

*Cryptocaryon irritans*, a species of obligatory ciliate ectoparasite, can infect various species of marine teleost fish. *Cryptocaryon irritans* that fall to the seabed or aquarium bottom in winter can form “dormant tomonts” and wake up when the temperature rises the next year. Abundant studies and analyses on the dormant tomonts were carried out at the transcriptome level, in order to investigate the molecular mechanism of *C. irritans* tomonts entering the dormant state under low-temperature conditions.

**Methods:**

The paired-end sequencing strategy was used to better assemble the entire transcriptome *de novo*. All clean sequencing reads from each of the three libraries (Group A: untreated blank control; Group B: treated for 24 h at 12 °C; and Group C: developed for 24 h at 25 °C) were respectively mapped back to the transcriptome assembly using the bioinformatics software.

**Results:**

In this study, 25,695,034, 21,944,467, and 28,722,875 paired-end clean reads were obtained respectively from the three cDNA libraries of the *C. irritans* tomont by Illumina paired-end sequencing technology. A total of 25,925 unique transcript fragments (unigenes) were assembled, with an average length of 839 bp. Differentially expressed genes (DEGs) were scrutinized; in Group B/A pairwise comparison, 343 genes presented differential expression, including 265 up-regulated genes and 78 down-regulated genes in Group B; in Group C/A pairwise comparison, there were 567 DEGs, including 548 up-regulated genes and 19 down-regulated genes in Group C; and in Group B/C pairwise comparison, 185 genes showed differential expression, including 145 up-regulated genes and 40 down-regulated genes in Group B.

**Conclusions:**

This is the first transcriptomic analytical study of the *C. irritans* tomonts under low temperature. It can be concluded that most of the genes required for its cell survival under low temperature, or for cell entry into a deeper dormancy state were discovered, and that they might be considered as candidate genes to develop the diagnostic and control measures for cryptocaryoniasis.

**Electronic supplementary material:**

The online version of this article (doi:10.1186/s13071-016-1550-1) contains supplementary material, which is available to authorized users.

## Background

Cryptocaryoniasis is categorized as a “Class II animal epidemic” in China, which is caused by the ciliate *Cryptocaryon irritans* inhabiting the body surface of marine teleosts. In recent years, this parasitic disease has often occurred in China’s coastal provinces and cities, bringing great losses to the fish farmers and relevant departments. In order to explore the pathogenesis of and effective control measures for *C. irritans*, researchers have carried out extensive studies from variable perspectives [1–3]. The regularity of parasite occurrence suggests that outbreaks of this disease are related to water temperature [4]. The incidence of this disease is relatively higher from April to November each year when the water temperature is between 20–30 °C than the rest of the year; after November, the incidence gradually reduces until complete disappearance; and after the 3–4-month low-temperature season, the disease would strike back again [5]. Researchers have revealed the 4-stage life-cycle of *C. irritans*: trophonts, protomonts, tomonts, and theronts. Trophonts mainly parasitize on the surfaces of the hosts, and leave their hosts after maturation, falling to seabed or aquarium bottom to form protomonts. Tomonts are formed after a brief phase of protomonts; and after continuous asymmetric division, 200–300 theronts are produced; theronts break the tomont wall and enter the water, swim very fast without food intake, and intrude into suitable hosts that they encounter, where they will develop into trophonts again [6]. Reports have proved that the development of the parasite is slow and can be even stopped at a low temperature. Tomonts can stay alive for 4–5 months at 12 °C. When water temperature rises, tomonts can wake up and start dividing again, producing infectious theronts. So it is believed that *C. irritans* that fall to seabed or aquarium bottom in winter can form “dormant tomonts” and wake up when the temperature rises next year [7].

Tomonts are a state of cells formed by ciliates and other protozoa after immobilizing from an active state, when they shrink gradually and lose some structures, followed by the formation of the tomont wall with secreted substances, forming a spherical or nearly spherical shape. Ciliate tomonts are classified into two types, namely dormant tomonts and proliferative tomonts; the former is a dormant state formed to withstand adverse environment, while the latter is a specific metamorphosis period of the life-cycle in which parasites in tomonts split into more daughter cells. It is therefore obvious that the formation and regulation of the two are different. Dormant tomonts are formed after a sudden change in temperature or food shortage, and they resume their normal activities after excystment once the environment is suitable. Current studies mainly focus on the processes of tomonts formation, dedifferentiation, and redifferentiation of free living ciliates [8]. The morphology of “proliferative tomonts” of *C. irritans* has also been described in great detail [1]. As for “dormant tomonts” described in this paper, however, they are formed when the cells stop dividing but keep alive after the water temperature decreases. This has been extensively investigated in other parasites and ciliates, e.g. *Colpoda maupasi* [9], *C. steinii* [10], dinoflagellate *Scrippsiella hangoei* [11], *Alexandrium catenella* and *A. tamarense* [12], etc. However, no study on the formation and regulation of dormant tomonts of *C. irritans* has been carried out yet.

A transcriptome represents all RNA transcripts in one cell or tissue, and reflects genes expressed in specific tissues in different life-cycle stages, physiological states, and environments [13]. Transcriptome studies can holistically exhibit functions and structures of genes and reveal the molecular mechanism of biological process and pathogenesis [14], thus transcriptomics has been widely applied in fundamental research, clinical diagnosis, drug development, and potential vaccine candidate proteins screening, etc. In recent years, RNA-sequencing has become a widely used approach in the studies on the development of ciliates, parasites, e.g. *Leishmania donovani* [15], salmon louse *Caligus rogercresseyi* [16]*,* and *Tetrahymena thermophila* [17], etc. For the studies on cryptocaryoniasis, Lokanathan et al. [18] generated and analyzed ESTs of *C. irritans* tomonts to identify genes that encode surface proteins, excretory/secretory proteins and repeat-containing proteins; and this is the only report so far. In the present study, tomonts were induced to enter the state of dormancy at 12 °C and the changes in transcriptome of dormant tomonts were compared with RNA-seq technology to explore the molecular mechanism of *C. irritans* tomonts entering dormant state in the low-temperature season.

## Methods

### *Cryptocaryon irritans* tomonts and collection

The *C. irritans* were derived from a naturally infected *Larimichthys crocea*, and *L. crocea* with an average body mass of 100 g were then used as animal models to establish the passage system [19]. The animal models were raised in a 1000 l aquarium (R × H: 60 × 60 cm), and were infected with a non-lethal concentration of theronts (≤ 10,000 theronts/fish) in 5 l of seawater per fish; 2 h after infection, fresh seawater was added. Four  days after infection, large numbers of tomonts were found to adhere to the bottom of aquarium. The fish were then transferred to another clean aquarium without tomonts and tomonts were collected by carefully discarding the debris and incubated in a 1 l beaker. Throughout the whole experiment, the water was oxygenated continuously and replaced to keep clean twice a day (09:00 and 15:00); the salinity, water temperature, light intensity, and photoperiod for aquaculture were 29–31 ‰, 26 ± 1 °C, 1000 lx, and 12 Light: 12 Dark, respectively.

Newly formed tomonts were collected within 10 h and divided into 3 groups: Group A, B and C. Group A was an untreated blank control group and placed in liquid nitrogen after drying; group B was treated for 24 h at 12 °C and placed in liquid nitrogen after drying; group C was set to develop normally for 24 h at room temperature (25 °C) without any extra-processing and placed in liquid nitrogen after drying. Previous studies have confirmed that at least a period of 36 h is required for newly formed tomonts to start dividing at about 25 °C, so the processing time in this study was set to 24 h to ensure that all cells should be about to divide but not yet. It was also confirmed microscopically that no cell division occurred in all three groups of tomonts described above before sampling.

### Total RNA extraction and sample preparation for RNA-Seq

Total RNA was extracted from the tomonts using an RNeasy Plus Universal Mini Kit (QIAGEN, Maryland, USA), which is specific for genome DNA elimination, according to the manufacturer’s instructions. All RNA was processed by RNase free DNase I provided by the kit. The quantity, purity and integrity of RNA were measured on a 1.2 % (w/v) agarose gel and on a Nanodrop-1000 spectrophotometer (NanoDrop, USA). Samples with higher quality (absorbance ratios at 260 nm/280 nm > 1.8) were selected for high-throughput sequencing. The extracted total RNA was resuspended in RNase free water and stored at -80 °C until use [20]. After enrichment with oligo-dT-attached magnetic beads, the purified mRNA was fragmented by divalent cations under elevated temperature, and then considered as a template for first-strand cDNA synthesis by random primers and reverse transcriptase. The second-strand cDNA was synthesized with RNase H (Invitrogen, USA) and DNA polymerase I. A paired-end library was constructed from the cDNA synthesized by the Genomic Sample Prep Kit (Illumina). Multiplexed cDNA libraries were mixed in equal volumes with normalized 10 nM concentration (Agilent 2100) [21, 22]. Three normalized cDNA libraries were constructed with the RNA from the control group, group B and group C. The library was sequenced by Illumina Mi-Seq platform.

### Assembly, comparative analysis and functional annotation of the transcriptome

To better assemble the entire transcriptome *de novo*, a paired-end (PE) sequencing strategy was used in this study. Raw PE reads with an average length of 250 bp were generated, and all sequences were examined for possible sequencing errors. Adaptor sequences and low quality sequences (i.e. the percentage of bases of quality value ≤ 5 exceeds 50 % in the read) were trimmed with the FastQC program (http://www.bioinformatics.babraham.ac.uk/projects/fastqc/). Short sequences (< 50 bp) were removed by a custom Perl program. The resulting high-quality sequences were *de novo* assembled into contigs and transcripts with Trinity software (https://github.com/trinityrnaseq/trinityrnaseq/wiki) [23]. To reduce data redundancy, transcripts with a minimum length of 200 bp were assembled and clustered by TGICL with default parameters. The longest sequences in each cluster were reserved and designated as unigenes [24]. All unigenes were used as queries in searching Nr, SwissProt and functionally annotated by GO analysis with Blast2GO software (E-value < 10^-5^) (https://www.blast2go.com/) [25]. KO analyses of differentially expressed unigenes (DEGs) in pathways were conducted on website (http://www.genome.jp/kegg/tool/map_pathway2.html). KEGG pathway analysis was performed using KEGG Automatic Annotation Server (KASS) with default parameters [26].

### Identification of DEGs

All clean sequencing reads from each of the three libraries (A, B and C) were, respectively, mapped back to the transcriptome assembly by the software Bowtie2 with default setting. The number of reads aligned to each unigene in the alignment file was counted for each sample. These read counts were normalized as RPKM (Reads per kilobase of transcripts per million fragments mapped) values [27] and further analysis of identify DEGs among different groups was conducted by a web tool DESeq (http://www-huber.embl.de/users/anders/DESeq). The *P*-value was applied to determine DEGs, and the FDR (false discovery rate) method was applied to determine the threshold *P*-value in multiple tests to judge the significance of the differences of gene expression. In our analysis, if the FDR was < 0.05 and RPKM values showing at least a 2-fold change among samples, these unigenes were considered as significant DEGs. Finally, DEGs were tested by GO functional analysis and KEGG pathway analysis [28].

## Results and discussion

### Sequencing and *de novo* transcriptome assembly

**Fig. 1:**
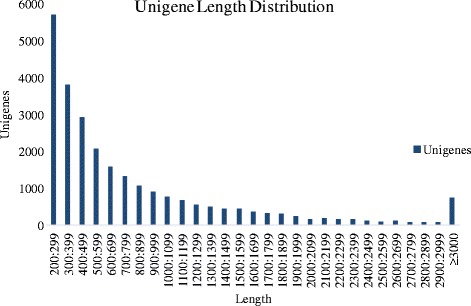
Transcriptome sequence length distributions of *C. irritans* tomont unigenes. The x-axis indicates unigene size and the y-axis indicates the number of unigenes with different lengths

To obtain the *C. irritans* tomont transcriptome expression profile during the dormant phase, the three cDNA libraries were constructed using tomonts from groups A, B and C. A total of 80,847,073 paired-end raw reads with an average length of 126 bp and a Q20 percentage higher than 96.02 % were generated (Additional file 1: Table S1). A total of 76,362,376 clean reads were obtained for subsequent analysis after eliminating low-quality sequences and adaptor sequences from the original data sequence by quality analysis (Additional file 1: Table S1). Transcript *de novo* assembly was performed for the clean reads by Trinity. A summary of all contigs, transcripts and unigenes assembly is presented in Table 1. The total length and number of contigs were 92,485,665 bp and 273,199, respectively. The maximum contig  length was 17,250 bp with an average length of 338.53 bp (N50:409), with the GC% of 46.05 %. The total length and number of transcripts were 84,205,775 bp and 162,496, respectively. The maximum length of transcript was 17,250 bp with an average length of 518 bp (N50:634), with GC% of 43.60 %. The total length and number of unigenes were 21,739,745 bp and 25,925, respectively. The maximal length of unigenes was 17,250 bp with an average length of 839 bp (N50:1250), and the GC% was 39.51 % (Table 1). Figure 1 demonstrated the distribution of the unigene lengths. The most abundant unigenes were clustered in a group with 200–299 bp in length. There were 742 unigenes with length of more than 3000 bp.

**Table 1:** Summary of the *de novo* assembly of transcriptomic profiles of *C. ittitans* tomonts

	Total length (bp)	No.	Max length(bp)	Average length (bp)	N50	GC %
Contig	92,485,665	273,199	17,250	338.53	409	46.05
Transcript	84,205,775	162,496	17,250	518	634	43.60
Unigene	21,739,745	25,925	17,250	839	1,250	39.51

### EggNOG

**Fig. 2:**
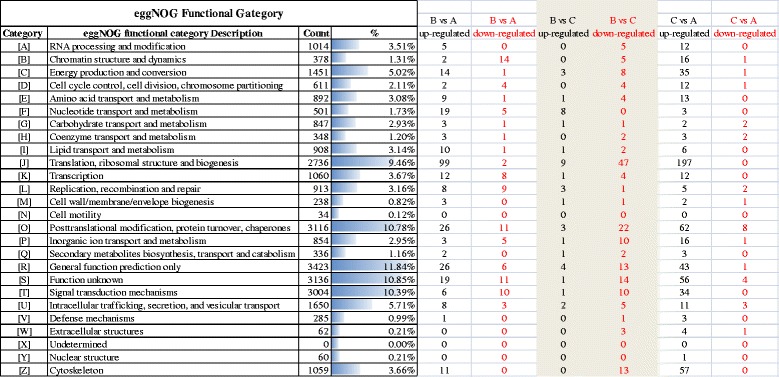
eggNOG function classification of *C. irritans* tomonts unigenes. A total of 23,670 hits were classified into 25 categories

For further functional prediction and classifications, unigenes were aligned to the eggNOG database. The eggNOG IDs of all genes were listed and these IDs were assigned into appropriated eggNOG categories, by which classified statistic was conducted to determine the functions of all genes, so as to learn the distribution patterns of gene functions of this species. A total of 23,670 (91.30 %) hits were annotated into 25,925 NR top hit unigenes (Table 2), which formed 25 classifications (Fig. 2). Among the functional classes, with the exception of the general function prediction only (3423, 11.84 %) and Function unknown (10.85 %), the largest three groups were post-translational modification, protein turnover and chaperones (10.78 %), signal transduction mechanisms (10.39 %), and translation, ribosomal structure and biogenesis (9.46 %). The smallest three groups were cell motility (0.12 %), nuclear structure (0.21 %), and extracellular structures (0.21 %) (Fig. 2).

**Table 2:** Annotation of unigenes of transcriptomic profiles of *C. irritans* tomonts

Database	Number of annotated unigenes	Percentage of annotated unigenes in NR top hit
Swiss-Prot	21,354	82.37
eggNOG	23,670	91.30
GO	21,510	82.97
KO	3,402	13.12
KEGG	6,607	25.49
NR top hit (Total)	25,925	100

Figure 2 presented the eggNOG analysis of DEGs from the three groups. In group B/A pairwise comparison, the up-regulated DEGs from eggNOG were functionally classified into 22 categories in which the three most enriched terms were J (99, Translation, ribosomal structure and biogenesis), O (26, Post-translational modification, protein turnover, chaperones) and R (26, General function prediction only); the down-regulated DEGs from eggNOG were functionally classified into 17 categories in which the three most enriched terms were B (14, Chromatin structure and dynamics), O (11) and S (11, Function unknown). In group B/C pairwise comparison, the up-regulated DEGs from eggNOG were functionally classified into 16 categories in which the 3 most enriched terms were the J (9), F (8, Nucleotide transport and metabolism), and R (4); the down-regulated DEGs from eggNOG were functionally classified into 22 categories in which the three most enriched terms were J (47), O (22), and S (14). In group C/A pairwise comparison, the regulated DEGs from eggNOG were functionally classified into 24 categories in which the three most enriched terms were J (197), O (62) and Z (57, Cytoskeleton); the down-regulated DEGs from eggNOG were functionally classified into 13 categories in which the three most enriched terms were O (8), S (4), and U (3, Intracellular trafficking, secretion and vesicular transport). It is found that some genes related to normal life activity such as protein synthesis regulations and cytoskeleton assembly (Fig. 2), were significantly inhibited by low-temperatures, which is different from ciliates originated from Antarctic region: the latter has developed a strong adaptability that allow tubulin post-translational modifications at low temperature [29].

### Identification of GO enrichment analysis

**Fig. 3:**
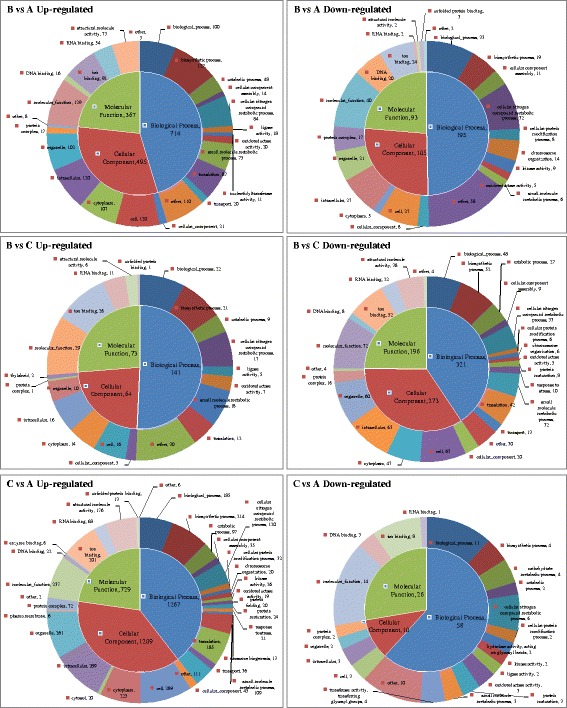
Gene ontology (GO) enrichment analysis of the differently expressed genes in pairwise comparison groups B *vs* A, B *vs* C and C* vs* A. A: untreated blank control, B: treated for 24 h at 12 °C, and C: developed for 24 h at 25 °C

Gene Ontology (GO) is a standardized gene functional classification system. In this study, a total of 21,510 unigenes (82.97 %) (Table 2) were assigned into three major functional GO terms (biological process category, cellular component category, and molecular function category), which were further summarized into 103 sub-categories using Blast2GO (Fig. 3).

Figure 3 represents pie charts of GO enrichment analysis of DEGs from above three groups. In C/A pairwise comparison, the up-regulated DEGs from GO were functionally classified into 61 sub-categories in which the three most enriched sub-categories were the cell (289, GO:0005623), intracellular (289, GO:0005622), and organelle (261, GO:0043226). The previous study on *C. irritans* tomont transcriptome indicated that proteins were annotated as the external encapsulating structure, cell projection proteins and proteinaceous extracellular matrix under the GO category cellular component were those with potential as serodiagnostic markers of the tomont stage parasites [18]. In groups B/A pairwise comparison, the up-regulated DEGs from GO were functionally classified into 56 sub-categories in which the three most enriched sub-categories were the molecular function (129, GO:0003674), biosynthetic process (127, GO:0009058), and cell (120); the down-regulated DEGs from GO were functionally classified into 48 sub-categories in which the three most enriched sub-categories were the molecular function (40), biological process (33, GO:0008150), and cellular nitrogen compound metabolic process (32, GO:0034641). In groups B/C pairwise comparison, the up-regulated DEGs from GO were functionally classified into 38 sub-categories in which the three most enriched sub-categories were the molecular function (29), ion binding (26, GO:0043167), and biological process (22); the down-regulated DEGs from GO were functionally classified into 48 sub-categories in which the three most enriched sub-categories were the molecular function (72), cell (63), intracellular (63). In a study on *Aurantiochytrium* sp. under cold stress, “cellular process”, “binding” and “metabolic process” were also the largest proportion sub-categories in the three categories, and fatty acid biosynthetic processes were also influenced [30]. The results above revealed the importance of biological processes and molecular function of the protozoans under low temperature.

### Functional classification by KEGG

**Fig. 4:**
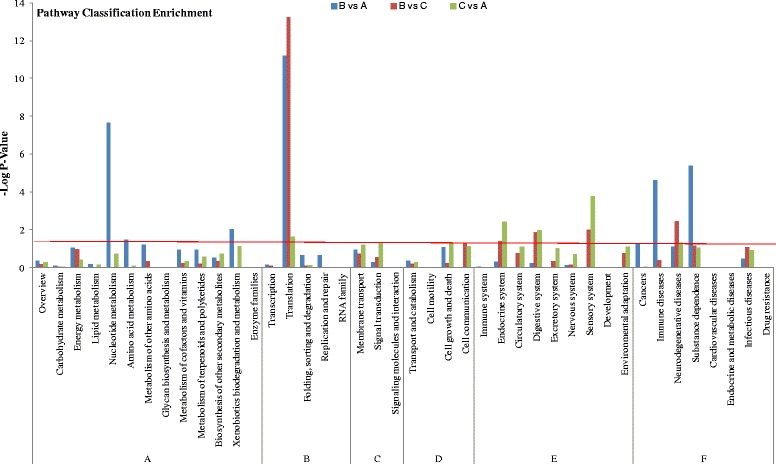
KEGG pathway enrichment analysis of the differently expressed genes in pairwise comparison groups B *vs* A, B *vs* C and C *vs* A. The x-axis represents KEGG pathway classification and y-axis represents the significance *P*-value of enrichment in hypergeometric distribution. The red line represents *P* = 0.05. A: Metabolism; B: Genetic information processing; C: Environmental information processing; D: Cellular processes; E: Organismal systems; F: Human diseases

To identify the biological pathways in *C. irritans* dormant tomonts, the unigenes were mapped to the reference pathways recorded in the KEGG database. A total of 6607 unigenes (25.49 %) (Table 2) were further annotated by KEGG and classified into six categories with 42 sub-classes (Fig. 4) (419 known KEGG pathways) (Additional files 2 and 3: Tables S2, S3).

Figure 4 shows the histogram of KEGG enrichment analysis of DEGs from above three groups. Compared with the whole genome expression, with -log_10_ (*P*-value) used to represent the enrichment degree, the most differential expressed enriched subclasses were as follows: Translation (B:A = 11.19*; C:A = 13.25*; C:B = 1.64*), Nucleotide metabolism (7.63*; 0.02; 0.72), Substance dependence (5.40*; 1.17; 1.05), Immune diseases (4.62*; 0.38; 0), Neurodegenerative diseases (1.11; 2.46*; 1.30*), Sensory system (0; 1.99*; 3.77*), Digestive system (0.24; 1.89*; 1.97*), Endocrine system (0.30; 1.37*; 2.41*), Xenobiotics biodegradation and metabolism (2.04*; 0; 1.14), Amino acid metabolism (1.49*; 0; 0.09), Signal transduction (0.25; 0.53; 1.34*), Cell communication (0; 1.33*; 1.14). “*” indicated significant difference. The ribosome pathway was the first of the most DEGs-enriched pathway (Additional file 3: Table S3). This result was similar to the results of the previous study on *C. irritans* tomont transcriptome [18].

**Fig. 5:**
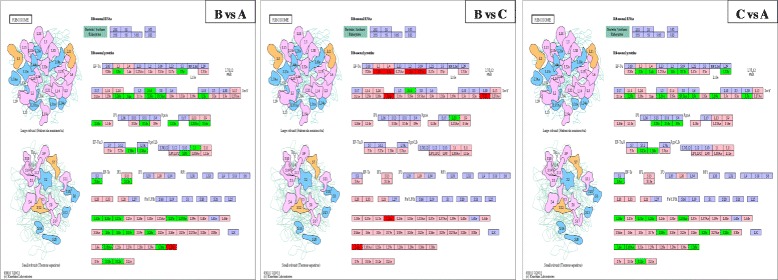
Gene list involved in ribosome pathway generated by KEGG of the DEGs in pairwise comparison groups B *vs* A, B *vs* C and C *vs* A. Green indicates significantly increased expression; red, significantly decreased expression; and pink, unchanged expression. Blue denotes genes that were not identified in the expression profile analysis

It can also be seen that although many genes encoding ribosomal proteins were up-regulated in both low temperature treated group (B, 63) and normally developed group (C, 139) compared with group A, the type and number of genes show great difference between groups B/A and groups C/A (Additional file 3: Table S3, Fig. 5). It is assumed that tomont cells under a different physiological condition would require different ribosomal proteins for the synthesis of a different set of proteins, and that these ribosomal proteins can also play various roles such as transcription, signal recognition, apoptosis and nuclear transport protein synthesis [18, 31].

### DEGs

**Fig. 6:**
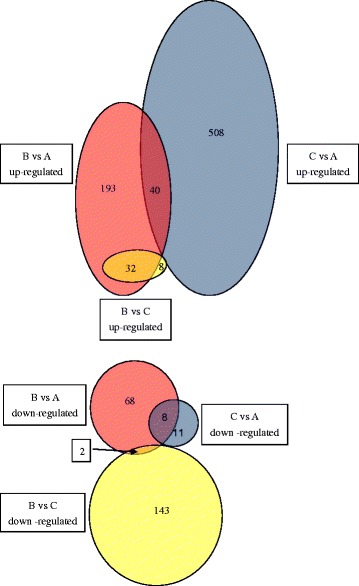
Pairwise comparisons of differentially expressed genes in pairwise comparison groups B *vs* A, B *vs* C and C *vs* A. The red, blue, and yellow circles indicate DEGs in groups B *vs *A, C *vs* A and B *vs* C, respectively

**Fig. 7:**
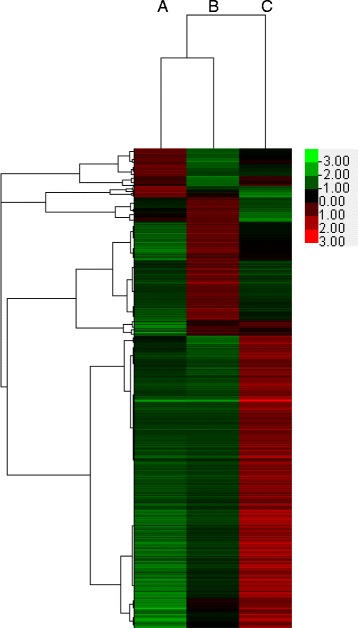
Heat map obtained from the hierarchical clustering of these genes. Shades of red to green represent the up- and down- regulation of genes within each row, where red denotes up-regulation and green denotes down-regulation

Water temperature influences the outbreak of cryptocaryoniasis, via promoting the growth or dormancy of *C. irritans* cells [4, 7]. To identify the DEGs involved in *C. irritans* response to low temperature treatment, pairwise comparisons for differential expression analysis were carried out among the three groups (A, B and C) (Fig. 6). Figure 7 demonstrates a heat map obtained from the hierarchical clustering of these genes.

In the group C/A pairwise comparison, there were 567 DEGs, including 548 up-regulated genes and 19 down-regulated genes in group C (Fig. 6, Additional file 4: Table S4 C/A). 96.65 % of the DEGs were up-regulated, indicating that despite absence of visible cell division in tomonts at this moment, numerous genes inside have been mobilized, preparing for cell division [32]. The top ten of DEGs identified as preferentially up-regulated at group C included *Tubulin/FtsZ family, XP_003884402.1, chaperone protein, collagen triple helix protein, AF466826_1histone H3p, Ribosomal protein L18, myo-inositol 1-phosphate synthase, Pyridoxal-dependent decarboxylase conserved domain containing protein, 60S ribosomal protein L10,* and *Ribosomal protein L37a*. The 19 down-regulated genes are presented in Additional file 4: Table S4 C/A. It is indicated that these 19 genes represent the remaining molecules [33], and may gradually become unnecessary as the cells approach division. Other proteins involved in the normal development of *C. irritans* tomonts will be described in detail in separate papers; in the present study these were mainly used to help identify proteins controlling tomont dormancy at low temperature.

In the group B/A pairwise comparison, 343 genes were differentially expressed in group B (Fig. 6, Additional file 4: Table S4 B/A columns A, L), i.e. 60.49 % (343/567) of these in the pairwise comparison group C/A, suggesting that tomonts of group B were still in a relatively active physiological condition after 24 h development at low temperature. This is because the tomonts were in a dynamic phase with rich internal life activities still going on instead of entering a fixed state immediately after the formation of tomonts. This was consistent with the study of Verni & Rosati [34]. However, most of the above DEGs (295/343) did not show significant differences in group C/A (Additional file 4: Table S4 B/A columns J, U), indicating a role of these genes in the low-temperature response, i.e. entering a dormant state.

Of all DEGs in the pairwise comparison group B/A, up to 77.26 % were up-regulated; of these, 40 were identical to the up-regulated DEGs in the pairwise comparison group C/A (Fig. 6, Additional file 4: Table S4 B/A), possibly involved in preparing for the restart of cell division and waking up when the water temperature rises again. Previous studies have confirmed that a mRNA pool appears in the dormant state of ciliates [35]. The stored mRNA such as actin, α-tubulin, HS70, glyceraldehydes-3-phosphate dehydrogenase, and metallothioneins in microbial cryptobiotic states might be required during the excystment process [33]. In the present study, the top ten out of 40 up-regulated DEGs included Ribosomal protein genes, *Histone H3*, and hypothetical and unnamed DEGs: *XP_003884402.1, XP_004036516.1, XP_001426579.1, CCF73045.1*. Among these DEGs, the highly expressed *Ribosomal protein L18 (related)* participates in stress adaptation by potentiating the cellular translation machinery to achieve a robust cytosolic stress response [36]. *60S ribosomal protein L10 (alpha/beta hammerhead)* genes are involved in development and translation under stress, such as ultraviolet B (UV-B) [37]. *Histone H3* was related to the aggregation of chromosomes [38]. *40S ribosomal protein* may be a subject to transcriptional regulation in different parasite life-cycle stages [39]; and *acidic ribosomal P0* protein is involved in cold-adaptation in ciliates [40]. Other up-regulated genes (Fig. 6, Additional file 4: Table S4 B/A) may be necessary for cells to survive at low temperature, or to enter a deeper level of dormancy. Of these, the highly expressed *ribosomal protein L7* could be involved in the suppression of global protein synthesis and the cell cycle under cold conditions [41]; *Bifunctional purine biosynthesis protein* (putative) catalyzes the second and the fifth steps of *de novo* purine biosynthesis pathway [42]; Vacuolar *ATP synthase* in *C. irritans* theronts with higher expression might be involved in the process of host infection [2]; *Heat shock protein 70* gene can improve protein folding together with some other chaperones [43]; *40S ribosomal protein S18* (putative) is involved in the initiation of translation [44]; *Glutamine synthetaseis* is involved in nitrogen metabolism, recycling of the neurotransmitter glutamate and glutamine biosynthesis for the production of amino acids, sugars, and glucosamine-6-phosphate [45]; *Eukaryotic initiation factor 4A* is a protein complex that mediates recruitment of ribosomes to mRNA [46]*; Ubiquitin-conjugating enzyme* (putative) is involved in selective protein degradation, DNA repair, cell cycle control, and possibly the regulation of chromatin structure [47]. The mechanism of these DEGs still requires further investigation, e.g. to determine the dynamic change in expression of the above-mentioned genes at different time points during low-temperature dormancy and waking up upon temperature rising.

We found that 78.38 % (145/185) of the DEGs were down-regulated in group B compared with group C (Fig. 6, Additional file 4: Table S4 B/C column L); of these, 135 (93.10 %) genes were up-regulated in pairwise comparison group C/A (Additional file 4: Table S4 B/C column T). In other words, all 548 up-regulated genes in group C/A (Additional file 4: Table S4 C/A column A) were down-regulated (135) or not significantly changed in group B/C (413) (Additional file 4: Table S4 C/A column I) suggesting that the genes associated with normal development and metabolism were significantly inhibited in group B after low-temperature treatment. This vital phenomenon, dormancy, is an important survival strategy in protozoans [34]. Every microbial cryptobiotic state is a direct consequence of the opening and closing of specific genes [33]. In the present study, the top ten down-regulated DEGs at group B/C included *Tubulin beta chain, Tubulin/FtsZ family, chaperone protein, cathepsin L, calmodulin, heat shock protein 70, EWS75591.1, cathepsin b, XP_003882889.1,* and *cut up CG6998-PA* (Additional file 4: Table S4). Tubulins are the major components of microtubule cytoskeletons in eukaryotic cells. Cytoskeleton is related to the morphological and structural changes of the cells, intracellular material transport, and dynamical system. In this study, Alpha/beta-tubulin and Tubulin/FtsZ family were highly up-regulated in the normally developed cells in group C because a large number of matrix proteins are essential for cells to form new cytoskeletons during cell division [2] but down-regulated by 999.28 and 590.19 times respectively in group B compared with group C. This confirmed that the activity associated with cell division was substantially inhibited. In addition, proteins associated with cytoskeleton assembly and regulation were also significantly down-regulated, such as *calmodulin, cut up CG6998-PA, dynein gamma chain (flagellar outer arm), and ciliary dynein heavy chain*, etc. And cathepsin is a type of protease related to the development, pathogenicity, as well as immune escape of the parasites [48].

In many ciliates, when environmental stress appears, this is identified by membrane receptors and opens a specific genetic programme that leads to dormant cyst formation [33]. In the group B/C pairwise comparison, eight of the 40 up-regulated genes were not significantly changed in group B compared with Group A (Additional file 4: Table S4 B/C column J), indicating a role in the regulation of tomonts entering a dormant state, including *Ion (cation) channel family protein* (ICFP)*, Succinate dehydrogenase iron-sulfur subunit* (SDIS)*, Triosephosphate isomerase* (TI)*, Malonyl coa-acyl carrier protein transacylase* (MCCPT)*, Ribosomal protein L12* (RPL12); *50S ribosomal protein L13* (RPL13)*, XP_001443344.1* and *XP_672154.1*. SDIS is a primary dehydrogenase and donates electrons to the aerobic and energy-generating respiratory chain in eukaryotic mitochondria and numerous prokaryotes [49]; TI is a crucial enzyme in the glycolytic pathway, which catalyzes the interconversion between dihydroxyacetone phosphate (DHAP) and D-glyceraldehyde 3-phosphate (GAP) by an isomerization reaction without any cofactor or metal ion [50]; MCCPT is one of the essential enzymes in the FAS II system [51]; RPL12 is required for the ribosome to function with *Elongation factor Tu, Initiation factor 2*, and *Reversing factor* [52]; RPL13 is essential for the formation of the RI*_50_ intermediate particle [53]. However, the dynamic changes and functions of these genes during low-temperature dormancy and recovery of tomonts remain to be further studied.

The previous study on *C. irritans* transcriptomic analysis identified a total of 690 unique transcripts encoding excretory/secretory proteins and membrane and membrane-associated proteins [18]; the proteomic analysis considered α-tubulin, actin, enolase, vacuolar ATP synthase catalytic subunit, and hsp70 potential vaccine antigen candidates [2]. In the present results, abundant known or unknown potential genes for vaccine development were provided (Additional file 4: Table S4), and further studies should be conducted to certify their functions.

## Conclusions

To our knowledge, this is the first transcriptomic study of *C. irritans* tomonts under low temperature conditions. It was concluded that most of the genes associated with normal cell development were inhibited at low temperature, but genes required for cell survival under low temperature, or for cell entry into a deeper dormancy state were significantly up-regulated. These genes might be considered as candidate genes to develop diagnostic and control measures for cryptocaryonosis. The results can serve as a reference for further studies on the mechanisms of low-temperature dormancy and recovery of parasites.

### Ethics statement

Experiments on fish and parasites were performed according to the regulations of local and central government. All experiments were approved by the Institutional Animal Care and Use Committee of the East China Sea Fisheries Research Institute, Chinese Academy of Fishery Sciences.

## Additional files

Additional file 1: Table S1.
*C. irritans* tomont transcriptome expression profile under low temperature. (DOCX 15 kb) Additional file 2: Table S2. KEGG pathway enrichment analysis of the differently expressed genes. (DOCX 40 kb) Additional file 3: Table S3. KEGG pathway enrichment analysis of the differently expressed genes among low temperature treated (B) and untreated (A, C) *C. irritans* tomonts. (XLSX 16 kb) Additional file 4: Table S4. Differently expressed genes among low temperature treated (B) and untreated (A, C) C. irritans tomonts. (XLSX 188 kb)
