# Supplementary material for: Transcriptome analysis of dormant tomonts of the marine fish ectoparasitic ciliate Cryptocaryon irritans under low temperature
Source: Parasit Vectors. 2016 May 13;9:280. doi: 10.1186/s13071-016-1550-1 (PMC4867990; doi:10.1186/s13071-016-1550-1)
Supplement: Additional file 1: Table S1. — C. irritans tomont transcriptome expression profile under low temperature. (DOCX 15 kb) [file 13071_2016_1550_MOESM1_ESM.docx]

**Table S1 *C. irritans* tomont transcriptome expression profile under low temperature**

| Statistics of raw data | | | | | |
| --- | --- | --- | --- | --- | --- |
| Sample | Reads | Raw Reads | Raw Data(bp) | Q20(%) | Reads Len.(bp) |
| A | R1 | 27,161,294 | 3,422,323,044 | 96.02 | 126 |
|  | R2 | 27,161,294 | 3,422,323,044 |  |  |
|  | Paired | 27,161,294 | 6,844,646,088 |  |  |
| B | R1 | 23,366,746 | 2,944,209,996 | 96.33 |  |
|  | R2 | 23,366,746 | 2,944,209,996 |  |  |
|  | Paired | 23,366,746 | 5,888,419,992 |  |  |
| C | R1 | 30,319,033 | 3,820,198,158 | 96.57 |  |
|  | R2 | 30,319,033 | 3,820,198,158 |  |  |
|  | Paired | 30,319,033 | 7,640,396,316 |  |  |
|  |  |  |  |  |  |
| Filtered Statistics (Q20) | | | | | |
| Sample | Reads | Clean Reads | Clean Data(bp) | Useful Reads % | Useful Data % |
| A | R1 | 25,695,034 | 3,139,114,914 | 94.60% | 92.37% |
|  | R2 | 25,695,034 | 3,182,945,664 |  |  |
|  | Paired | 25,695,034 | 6,322,060,578 |  |  |
| B | R1 | 21,944,467 | 2,703,157,420 | 93.91% | 91.85% |
|  | R2 | 21,944,467 | 2,705,153,858 |  |  |
|  | Paired | 21,944,467 | 5,408,311,278 |  |  |
| C | R1 | 28,722,875 | 3,527,779,138 | 94.74% | 92.58% |
|  | R2 | 28,722,875 | 3,546,048,438 |  |  |
|  | Paired | 28,722,875 | 7,073,827,576 |  |  |
