# Supplementary material for: Transcriptome analysis of dormant tomonts of the marine fish ectoparasitic ciliate Cryptocaryon irritans under low temperature
Source: Parasit Vectors. 2016 May 13;9:280. doi: 10.1186/s13071-016-1550-1 (PMC4867990; doi:10.1186/s13071-016-1550-1)
Supplement: Additional file 2: Table S2. — KEGG pathway enrichment analysis of the differently expressed genes. (DOCX 40 kb) [file 13071_2016_1550_MOESM2_ESM.docx]

**Table S2 KEGG pathway enrichment analysis of the differently expressed genes**

| **Metabolism** | | Gene No. |
| --- | --- | --- |
| **Overview** | |  |
| ko01200 | Carbon metabolism | 112 |
| ko01210 | 2-Oxocarboxylic acid metabolism | 20 |
| ko01212 | Fatty acid metabolism | 33 |
| ko01230 | Biosynthesis of amino acids | 87 |
| ko01220 | Degradation of aromatic compounds | 2 |
| **Carbohydrate metabolism** | |  |
| ko00010 | Glycolysis / Gluconeogenesis | 44 |
| ko00020 | Citrate cycle (TCA cycle) | 48 |
| ko00030 | Pentose phosphate pathway | 24 |
| ko00040 | Pentose and glucuronate interconversions | 5 |
| ko00051 | Fructose and mannose metabolism | 18 |
| ko00052 | Galactose metabolism | 13 |
| ko00053 | Ascorbate and aldarate metabolism | 6 |
| ko00500 | Starch and sucrose metabolism | 20 |
| ko00520 | Amino sugar and nucleotide sugar metabolism | 32 |
| ko00620 | Pyruvate metabolism | 42 |
| ko00630 | Glyoxylate and dicarboxylate metabolism | 30 |
| ko00640 | Propanoate metabolism | 20 |
| ko00650 | Butanoate metabolism | 12 |
| ko00562 | Inositol phosphate metabolism | 24 |
| **Energy metabolism** | |  |
| ko00190 | Oxidative phosphorylation | 88 |
| ko00195 | Photosynthesis | 1 |
| ko00710 | Carbon fixation in photosynthetic organisms | 30 |
| ko00720 | Carbon fixation pathways in prokaryotes | 17 |
| ko00680 | Methane metabolism | 21 |
| ko00910 | Nitrogen metabolism | 12 |
| ko00920 | Sulfur metabolism | 12 |
| **Lipid metabolism** | |  |
| ko00061 | Fatty acid biosynthesis | 11 |
| ko00062 | Fatty acid elongation | 11 |
| ko00071 | Fatty acid degradation | 21 |
| ko00072 | Synthesis and degradation of ketone bodies | 5 |
| ko00073 | Cutin, suberine and wax biosynthesis | 4 |
| ko00100 | Steroid biosynthesis | 8 |
| ko00120 | Primary bile acid biosynthesis | 3 |
| ko00140 | Steroid hormone biosynthesis | 4 |
| ko00561 | Glycerolipid metabolism | 17 |
| ko00564 | Glycerophospholipid metabolism | 38 |
| ko00565 | Ether lipid metabolism | 12 |
| ko00600 | Sphingolipid metabolism | 8 |
| ko00590 | Arachidonic acid metabolism | 11 |
| ko00591 | Linoleic acid metabolism | 1 |
| ko00592 | alpha-Linolenic acid metabolism | 4 |
| ko01040 | Biosynthesis of unsaturated fatty acids | 11 |
| **Nucleotide metabolism** | |  |
| ko00230 | Purine metabolism | 114 |
| ko00240 | Pyrimidine metabolism | 96 |
| **Amino acid metabolism** | |  |
| ko00250 | Alanine, aspartate and glutamate metabolism | 29 |
| ko00260 | Glycine, serine and threonine metabolism | 27 |
| ko00270 | Cysteine and methionine metabolism | 28 |
| ko00280 | Valine, leucine and isoleucine degradation | 27 |
| ko00290 | Valine, leucine and isoleucine biosynthesis | 2 |
| ko00300 | Lysine biosynthesis | 8 |
| ko00310 | Lysine degradation | 20 |
| ko00330 | Arginine and proline metabolism | 29 |
| ko00340 | Histidine metabolism | 8 |
| ko00350 | Tyrosine metabolism | 13 |
| ko00360 | Phenylalanine metabolism | 9 |
| ko00380 | Tryptophan metabolism | 18 |
| ko00400 | Phenylalanine, tyrosine and tryptophan biosynthesis | 10 |
| **Metabolism of other amino acids** | |  |
| ko00410 | beta-Alanine metabolism | 10 |
| ko00430 | Taurine and hypotaurine metabolism | 1 |
| ko00440 | Phosphonate and phosphinate metabolism | 6 |
| ko00450 | Selenocompound metabolism | 11 |
| ko00460 | Cyanoamino acid metabolism | 2 |
| ko00471 | D-Glutamine and D-glutamate metabolism | 2 |
| ko00480 | Glutathione metabolism | 38 |
| **Glycan biosynthesis and metabolism** | |  |
| ko00510 | N-Glycan biosynthesis | 27 |
| ko00513 | Various types of N-glycan biosynthesis | 12 |
| ko00512 | Mucin type O-glycan biosynthesis | 1 |
| ko00514 | Other types of O-glycan biosynthesis | 1 |
| ko00531 | Glycosaminoglycan degradation | 1 |
| ko00563 | Glycosylphosphatidylinositol(GPI)-  anchor biosynthesis | 11 |
| ko00603 | Glycosphingolipid biosynthesis - globo series | 2 |
| ko00604 | Glycosphingolipid biosynthesis - ganglio series | 2 |
| ko00540 | Lipopolysaccharide biosynthesis | 1 |
| ko00511 | Other glycan degradation | 1 |
| **Metabolism of cofactors and vitamins** | |  |
| ko00730 | Thiamine metabolism | 6 |
| ko00740 | Riboflavin metabolism | 4 |
| ko00750 | Vitamin B6 metabolism | 4 |
| ko00760 | Nicotinate and nicotinamide metabolism | 11 |
| ko00770 | Pantothenate and CoA biosynthesis | 9 |
| ko00780 | Biotin metabolism | 6 |
| ko00785 | Lipoic acid metabolism | 2 |
| ko00790 | Folate biosynthesis | 14 |
| ko00670 | One carbon pool by folate | 6 |
| ko00830 | Retinol metabolism | 4 |
| ko00860 | Porphyrin and chlorophyll metabolism | 18 |
| ko00130 | Ubiquinone and other terpenoid-quinone biosynthesis | 5 |
| **Metabolism of terpenoids and polyketides** | |  |
| ko00900 | Terpenoid backbone biosynthesis | 27 |
| ko00909 | Sesquiterpenoid and triterpenoid biosynthesis | 2 |
| ko00908 | Zeatin biosynthesis | 4 |
| ko00903 | Limonene and pinene degradation | 3 |
| ko00281 | Geraniol degradation | 4 |
| ko01051 | Biosynthesis of ansamycins | 3 |
| **Biosynthesis of other secondary metabolites** | |  |
| ko00940 | Phenylpropanoid biosynthesis | 1 |
| ko00901 | Indole alkaloid biosynthesis | 1 |
| ko00950 | Isoquinoline alkaloid biosynthesis | 5 |
| ko00960 | Tropane, piperidine and pyridine alkaloid biosynthesis | 4 |
| ko00965 | Betalain biosynthesis | 1 |
| ko00521 | Streptomycin biosynthesis | 10 |
| ko00524 | Butirosin and neomycin biosynthesis | 3 |
| ko00401 | Novobiocin biosynthesis | 2 |
| ko00254 | Aflatoxin biosynthesis | 1 |
| **Xenobiotics biodegradation and metabolism** | |  |
| ko00362 | Benzoate degradation | 7 |
| ko00627 | Aminobenzoate degradation | 6 |
| ko00364 | Fluorobenzoate degradation | 1 |
| ko00625 | Chloroalkane and chloroalkene degradation | 4 |
| ko00361 | Chlorocyclohexane and chlorobenzene degradation | 1 |
| ko00623 | Toluene degradation | 1 |
| ko00622 | Xylene degradation | 1 |
| ko00643 | Styrene degradation | 3 |
| ko00930 | Caprolactam degradation | 4 |
| ko00621 | Dioxin degradation | 1 |
| ko00626 | Naphthalene degradation | 3 |
| ko00980 | Metabolism of xenobiotics by cytochrome P450 | 8 |
| ko00982 | Drug metabolism - cytochrome P450 | 7 |
| ko00983 | Drug metabolism - other enzymes | 15 |
|  |  |  |
| **Genetic Information Processing** | |  |
| **Transcription** | |  |
| ko03020 | RNA polymerase | 32 |
| ko03022 | Basal transcription factors | 15 |
| ko03040 | Spliceosome | 150 |
| **Translation** | |  |
| ko03010 | Ribosome | 357 |
| ko00970 | Aminoacyl-tRNA biosynthesis | 58 |
| ko03013 | RNA transport | 104 |
| ko03015 | mRNA surveillance pathway | 53 |
| ko03008 | Ribosome biogenesis in eukaryotes | 100 |
| **Folding, sorting and degradation** | |  |
| ko03060 | Protein export | 32 |
| ko04141 | Protein processing in endoplasmic reticulum | 104 |
| ko04130 | SNARE interactions in vesicular transport | 14 |
| ko04120 | Ubiquitin mediated proteolysis | 75 |
| ko04122 | Sulfur relay system | 12 |
| ko03050 | Proteasome | 66 |
| ko03018 | RNA degradation | 68 |
| **Replication and repair** | |  |
| ko03030 | DNA replication | 40 |
| ko03410 | Base excision repair | 29 |
| ko03420 | Nucleotide excision repair | 39 |
| ko03430 | Mismatch repair | 25 |
| ko03440 | Homologous recombination | 20 |
| ko03450 | Non-homologous end-joining | 11 |
| ko03460 | Fanconi anemia pathway | 20 |
|  |  |  |
| **Environmental Information Processing** | |  |
| **Membrane transport** | |  |
| ko02010 | ABC transporters | 6 |
| ko03070 | Bacterial secretion system | 3 |
| **Signal transduction** | |  |
| ko02020 | Two-component system | 23 |
| ko04014 | Ras signaling pathway | 22 |
| ko04015 | Rap1 signaling pathway | 13 |
| ko04010 | MAPK signaling pathway | 27 |
| ko04013 | MAPK signaling pathway - fly | 5 |
| ko04011 | MAPK signaling pathway - yeast | 2 |
| ko04012 | ErbB signaling pathway | 11 |
| ko04310 | Wnt signaling pathway | 28 |
| ko04330 | Notch signaling pathway | 9 |
| ko04340 | Hedgehog signaling pathway | 11 |
| ko04350 | TGF-beta signaling pathway | 17 |
| ko04390 | Hippo signaling pathway | 24 |
| ko04391 | Hippo signaling pathway -fly | 19 |
| ko04370 | VEGF signaling pathway | 14 |
| ko04630 | Jak-STAT signaling pathway | 2 |
| ko04064 | NF-kappa B signaling pathway | 8 |
| ko04668 | TNF signaling pathway | 10 |
| ko04066 | HIF-1 signaling pathway | 28 |
| ko04068 | FoxO signaling pathway | 39 |
| ko04020 | Calcium signaling pathway | 29 |
| ko04070 | Phosphatidylinositol signaling system | 28 |
| ko04151 | PI3K-Akt signaling pathway | 59 |
| ko04150 | mTOR signaling pathway | 28 |
| **Signaling molecules and interaction** | |  |
| ko04080 | Neuroactive ligand-receptor interaction | 3 |
|  |  |  |
| **Cellular Processes** | |  |
| **Transport and catabolism** | |  |
| ko04144 | Endocytosis | 56 |
| ko04145 | Phagosome | 60 |
| ko04142 | Lysosome | 67 |
| ko04146 | Peroxisome | 49 |
| ko04140 | Regulation of autophagy | 19 |
| **Cell motility** | |  |
| ko04810 | Regulation of actin cytoskeleton | 22 |
| **Cell growth and death** | |  |
| ko04110 | Cell cycle | 67 |
| ko04111 | Cell cycle - yeast | 65 |
| ko04112 | Cell cycle - Caulobacter | 4 |
| ko04113 | Meiosis - yeast | 51 |
| ko04114 | Oocyte meiosis | 67 |
| ko04210 | Apoptosis | 18 |
| ko04115 | p53 signaling pathway | 14 |
| **Cell communication** | |  |
| ko04510 | Focal adhesion | 15 |
| ko04520 | Adherens junction | 10 |
| ko04530 | Tight junction | 19 |
| ko04540 | Gap junction | 18 |
|  |  |  |
| **Organismal Systems** | |  |
| **Immune system** | |  |
| ko04611 | Platelet activation | 15 |
| ko04620 | Toll-like receptor signaling pathway | 8 |
| ko04621 | NOD-like receptor signaling pathway | 11 |
| ko04622 | RIG-I-like receptor signaling pathway | 4 |
| ko04623 | Cytosolic DNA-sensing pathway | 16 |
| ko04650 | Natural killer cell mediated cytotoxicity | 14 |
| ko04612 | Antigen processing and presentation | 22 |
| ko04660 | T cell receptor signaling pathway | 14 |
| ko04662 | B cell receptor signaling pathway | 15 |
| ko04664 | Fc epsilon RI signaling pathway | 8 |
| ko04666 | Fc gamma R-mediated phagocytosis | 24 |
| ko04670 | Leukocyte transendothelial migration | 3 |
| ko04062 | Chemokine signaling pathway | 15 |
| **Endocrine system** | |  |
| ko04911 | Insulin secretion | 8 |
| ko04910 | Insulin signaling pathway | 44 |
| ko04920 | Adipocytokine signaling pathway | 17 |
| ko03320 | PPAR signaling pathway | 14 |
| ko04912 | GnRH signaling pathway | 19 |
| ko04913 | Ovarian Steroidogenesis | 4 |
| ko04915 | Estrogen signaling pathway | 27 |
| ko04914 | Progesterone-mediated oocyte maturation | 40 |
| ko04917 | Prolactin signaling pathway | 8 |
| ko04921 | Oxytocin signaling pathway | 37 |
| ko04918 | Thyroid hormone synthesis | 18 |
| ko04919 | Thyroid hormone signaling pathway | 24 |
| ko04916 | Melanogenesis | 15 |
| ko04614 | Renin-angiotensin system | 2 |
| **Circulatory system** | |  |
| ko04260 | Cardiac muscle contraction | 14 |
| ko04261 | Adrenergic signaling in cardiomyocytes | 33 |
| ko04270 | Vascular smooth muscle contraction | 18 |
| **Digestive system** | |  |
| ko04970 | Salivary secretion | 20 |
| ko04971 | Gastric acid secretion | 11 |
| ko04972 | Pancreatic secretion | 13 |
| ko04976 | Bile secretion | 8 |
| ko04973 | Carbohydrate digestion and absorption | 3 |
| ko04974 | Protein digestion and absorption | 2 |
| ko04975 | Fat digestion and absorption | 4 |
| ko04977 | Vitamin digestion and absorption | 1 |
| ko04978 | Mineral absorption | 2 |
| **Excretory system** | |  |
| ko04962 | Vasopressin-regulated water reabsorption | 26 |
| ko04960 | Aldosterone-regulated sodium reabsorption | 4 |
| ko04961 | Endocrine and other factor-regulated  calcium reabsorption | 13 |
| ko04964 | Proximal tubule bicarbonate reclamation | 7 |
| ko04966 | Collecting duct acid secretion | 23 |
| **Nervous system** | |  |
| ko04724 | Glutamatergic synapse | 21 |
| ko04727 | GABAergic synapse | 15 |
| ko04725 | Cholinergic synapse | 13 |
| ko04728 | Dopaminergic synapse | 34 |
| ko04726 | Serotonergic synapse | 12 |
| ko04720 | Long-term potentiation | 23 |
| ko04730 | Long-term depression | 20 |
| ko04723 | Retrograde endocannabinoid signaling | 8 |
| ko04721 | Synaptic vesicle cycle | 46 |
| ko04722 | Neurotrophin signaling pathway | 23 |
| **Sensory system** | |  |
| ko04744 | Phototransduction | 4 |
| ko04745 | Phototransduction - fly | 4 |
| ko04740 | Olfactory transduction | 12 |
| ko04742 | Taste transduction | 6 |
| ko04750 | Inflammatory mediator regulation of TRP channels | 13 |
| **Development** | |  |
| ko04320 | Dorso-ventral axis formation | 7 |
| ko04360 | Axon guidance | 15 |
| ko04380 | Osteoclast differentiation | 13 |
| **Environmental adaptation** | |  |
| ko04710 | Circadian rhythm | 14 |
| ko04713 | Circadian entrainment | 16 |
| ko04711 | Circadian rhythm - fly | 3 |
| ko04712 | Circadian rhythm - plant | 6 |
| ko04626 | Plant-pathogen interaction | 27 |
|  |  |  |
| **Human Diseases** | |  |
| **Cancers** | |  |
| ko05200 | Pathways in cancer | 41 |
| ko05202 | Transcriptional misregulation in cancers | 12 |
| ko05206 | MicroRNAs in cancer | 10 |
| ko05205 | Proteoglycans in cancer | 28 |
| ko05204 | Chemical carcinogenesis | 8 |
| ko05203 | Viral carcinogenesis | 56 |
| ko05210 | Colorectal cancer | 18 |
| ko05212 | Pancreatic cancer | 10 |
| ko05214 | Glioma | 14 |
| ko05216 | Thyroid cancer | 5 |
| ko05221 | Acute myeloid leukemia | 8 |
| ko05220 | Chronic myeloid leukemia | 10 |
| ko05217 | Basal cell carcinoma | 3 |
| ko05218 | Melanoma | 9 |
| ko05211 | Renal cell carcinoma | 11 |
| ko05219 | Bladder cancer | 7 |
| ko05215 | Prostate cancer | 20 |
| ko05213 | Endometrial cancer | 11 |
| ko05222 | Small cell lung cancer | 14 |
| ko05223 | Non-small cell lung cancer | 11 |
| **Immune diseases** | |  |
| ko05322 | Systemic lupus erythematosus | 27 |
| ko05323 | Rheumatoid arthritis | 34 |
| ko05340 | Primary immunodeficiency | 4 |
| **Neurodegenerative diseases** | |  |
| ko05010 | Alzheimer's disease | 70 |
| ko05012 | Parkinson's disease | 67 |
| ko05014 | Amyotrophic lateral sclerosis (ALS) | 16 |
| ko05016 | Huntington's disease | 109 |
| ko05020 | Prion diseases | 16 |
| **Substance dependence** | |  |
| ko05030 | Cocaine addiction | 5 |
| ko05031 | Amphetamine addiction | 16 |
| ko05032 | Morphine addiction | 8 |
| ko05034 | Alcoholism | 41 |
| **Cardiovascular diseases** | |  |
| ko05410 | Hypertrophic cardiomyopathy (HCM) | 8 |
| ko05414 | Dilated cardiomyopathy (DCM) | 4 |
| ko05416 | Viral myocarditis | 8 |
| **Endocrine and metabolic diseases** | |  |
| ko04940 | Type I diabetes mellitus | 1 |
| ko04930 | Type II diabetes mellitus | 8 |
| ko04932 | Non-alcoholic fatty liver disease (NAFLD) | 52 |
| **Infectious diseases** | |  |
| ko05110 | Vibrio cholerae infection | 43 |
| ko05120 | Epithelial cell signaling in  Helicobacter pylori infection | 33 |
| ko05130 | Pathogenic Escherichia coli infection | 11 |
| ko05132 | Salmonella infection | 18 |
| ko05131 | Shigellosis | 11 |
| ko05133 | Pertussis | 9 |
| ko05134 | Legionellosis | 32 |
| ko05152 | Tuberculosis | 44 |
| ko05100 | Bacterial invasion of epithelial cells | 10 |
| ko05166 | HTLV-I infection | 63 |
| ko05162 | Measles | 26 |
| ko05164 | Influenza A | 37 |
| ko05161 | Hepatitis B | 18 |
| ko05160 | Hepatitis C | 27 |
| ko05168 | Herpes simplex infection | 38 |
| ko05169 | Epstein-Barr virus infection | 111 |
| ko05146 | Amoebiasis | 8 |
| ko05145 | Toxoplasmosis | 21 |
| ko05140 | Leishmaniasis | 2 |
| ko05142 | Chagas disease (American trypanosomiasis) | 14 |
| ko05143 | African trypanosomiasis | 1 |
